# Long-term epilepsy associated-tumors (LEATs): what is new?

**DOI:** 10.1055/s-0043-1777730

**Published:** 2023-12-29

**Authors:** Sergio Rosemberg

**Affiliations:** 1 Universidade de São Paulo, Faculdade de Medicina, Departamento de Patologia, São Paulo SP, Brazil.; 2 Santa Casa de São Paulo, Faculdade de Ciências Médicas, São Paulo SP, Brazil.

**Keywords:** Epilepsy, Brain Neoplasms, Astrocytoma, Neoplasms, Neuroepithelial, Epilepsia, Neoplasias Encefálicas, Astrocitoma, Neoplasias Neuroepiteliomatosas

## Abstract

Long-term epilepsy-associated tumors (LEATs) include a series of neoplasms that commonly occur in children, adolescents, or young adults, have an astrocytic or glioneuronal lineage, are histologically benign (WHO grade1) with a neocortical localization predominantly situated in the temporal lobes. Clinically, chronic refractory epilepsy is usually the unique symptom. Gangliogliomas (GG) and dysembryoplastic neuroepithelial tumors (DNT) are the most common representative entities besides pilocytic astrocytomas (PA) and angiocentric gliomas (AG). Recent molecular studies have defined new clinicopathological entities, which are recognized by the WHO 2021 classification of brain tumors. Some of them such as diffuse astrocytoma *MIB* or *MYBL1* altered, polymorphous low-grade neuroepithelial tumor of the young (PLNTY), and multilocular and vacuolating neuronal tumor (MVNT) are currently considered LEATs. The relationship between LEATs and epilepsy is still a matter of debate, and there is a general agreement about the beneficial effects of an early neurosurgical intervention on the clinical outcome.

## Resumo

Tumores associados a epilepsia de longa duração constituem uma série de neoplasias asatrocitárias ou glioneuronais que comumente incidem em crianças, adolescentes e jovens adultos e que são histologicamente benignos (OMS grau 1), de localização neocortical e predominantemente situados nos lobos temporais. Clinicamente, a epilepsia crônica refratária é, de modo geral, o único sintoma. Gangliogliomas (GG) e tumores neuroepiteliais disembrioplásticos (DNT) são as entidades mais representativas associadas a astrocitomas pilocíticos (AP) e gliomas angiocêntricos (GA). Estudos moleculares recentes permitiram a definição de novas entidades clínico-patológicas reconhecidas pela classificação de tumores cerebrais da OMS 2021. Algumas delas, como o astrocitoma difuso *MIB* ou *MIBL1* alterados, o tumor neuroepitelial polimorfo do jovem (PLNTY) e o tumor neuronal multilocular e vacuolizado (MVNT) são atualmente considerados tumores associados a epilepsia de longa duração. A relação entre este grupo de tumores e epilepsia é ainda debatida e há um consenso geral sobre o benefício prognóstico de intervenção cirúrgica precoce.

## INTRODUCTION

Since the classical studies of Hughlings Jackson at the end of the XIX century, the incidence of epilepsy induced by brain tumors has been a well-known phenomenon. However, in the last two or three decades, after the advent and popularization of MRI and more sophisticated electrophysiological methods, it occurred a worldwide spreading of surgery for patients presenting a long-standing history of chronic seizures. As a consequence, it became evident that three main pathologic conditions are responsible for the vast majority of the cases. Of these, hippocampal sclerosis (HS) is by far the most common in adults whereas focal cortical dysplasia (FCD) and a very special cohort of tumors are found in pediatric and young patients. 1 Luyken et al. in 2003 2 coined the term “long-term epilepsy-associated tumors (LEATs)” for these neoplasms. LEATs are distinguished from conventional brain tumors by a young age of onset of symptoms (with epilepsy usually as the primary and often the only neurological symptom), slow growth, neocortical localization, and often, a temporal lobe predominance. 3 In their series, children who underwent surgery have been investigated and treated for drug-resistant epilepsy for two years or longer.

## CONCEPT AND DEFINITION

In the different series dedicated to the management of chronic refractory epilepsy by neurosurgical approach, these “LEATs” included a variable number of histopathological entities. All of them were low-grade (1-2) neuroepithelial tumors. Glioneuronal tumors such as gangliogliomas (GG) and dysembryoplastic neuroepithelial tumors (DNT) were the most frequent, followed by astrocytic gliomas such as pilocytic astrocytomas (PA), and pleomorphic xanthoastrocytoma (PXA). Diffuse low-grade (grade 2) gliomas as astrocytomas and oligodendrogliomas could be also associated with long-standing epilepsy.

Nowadays, the concept of LEATs is more restrictive. This term should define, as mentioned above, a group of neuroepithelial neoplasms bearing the characteristics as follows:

- precocity of symptoms (mainly in the two first decades);
- clinically traduced only by epileptic seizures generally focal;
- lack of other neurological signs or symptoms such as intracranial hypertension, motor or sensitive disturbances, etc.;
- discrete (rarely more diffuse) cortical localization;
- histopathologically benign (grade 1).

In this sense, some tumors classically considered LEATs, such as PXA, papillary glioneuronal tumor (PGNT), or rosette-forming glioneuronal tumor (RFGT) may not fulfill those items and should not be included among them. Indeed, in some recent manuscripts 4 they are ruled out the table concerning these neoplasms.

Recently, the term “LEAT” has been challenged by some authors. 5 6 Indeed, for reasons discussed below, neurosurgical management for these tumors has been performed as precociously as possible making the terms “long-term” or “chronic” epilepsy no longer justified. As all LEATs as defined above are benign neuroepithelial tumors, the term “low-grade epilepsy-associated neuroepithelial tumors” has been proposed and has gained acceptance in the modern literature. 7 8

## HISTOPATHOLOGY

The classification of the entities discussed herewith follows the WHO Central Nervous System Tumours 2021. 9 In this classification, besides the classical histological parameter to define a diagnosis, molecular and genetic analysis were introduced, 10 so that new entities became recognized, some of them are currently considered LEATs.

### Ganglioglioma (GG)

**Figure 1:**
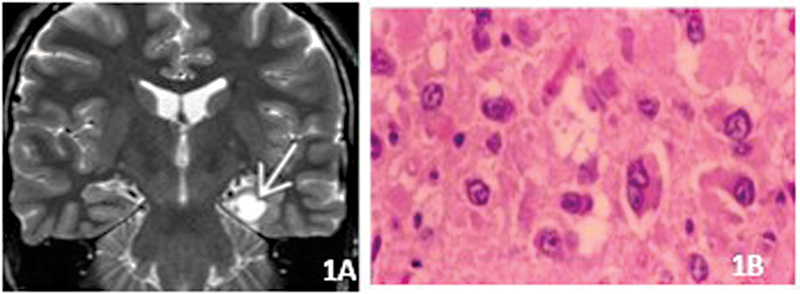
Ganglioglioma A) Coronal T2. Discrete lesion, without oedema or mass effect. B) Admixture of neurons and glial cell. Note binucleated neuron.

This is by far the most frequent of LEATs (5). About 70% are located in the temporal lobes. 11 Most occur in the first two decades of life. At MRI, commonly it is represented by a discrete nodular mass T1-hypointense, T2 hyperintense, which may be associated with a cystic component without mass effect ( Figure 1A ). Contrast enhancement is variable. Histologically, it is a biphasic tumor composed of an admixture of astrocytes and neurons ( Figure 1B ). GG is a grade 1 neoplasm. However, there are several reports on malignant transformation (anaplastic gangliogliomas). 11 12 Nevertheless, as stated by Slegers et al. 13 most of the prior studies lacked molecular analysis to exclude other high-grade gliomas subtypes, a fact that claims for further studies to confirm the existence of this phenomenon. Between one-third to half of the GG harbor BRAF ^V600E^ alterations.

### Dysembrioplastic neuroepithelial tumor (DNT)

**Figure 2:**
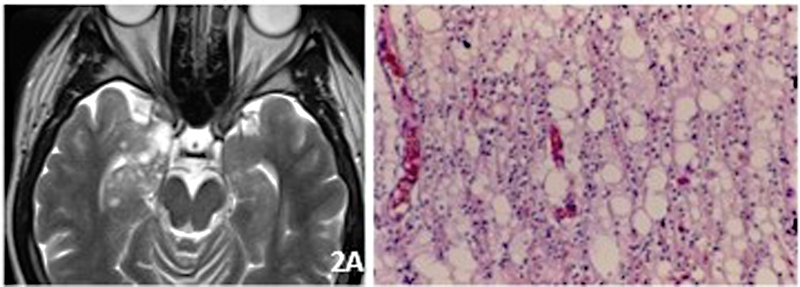
DNT. A) Axial T2. Microcysts, “soap-bubble” aspect. B) Columns of oligo-like cells, mucoid matrix and “floating” neurons.

This is the second most frequent LEAT in all series. It is located in the temporal lobes in about 2/3 of the cases. 14 Epilepsy starts in childhood or adolescence. It is situated almost entirely at the cortex and commonly it exhibits a lobulated architecture. On MRI it is T1 iso or hypointense, T2/flair hyperintense, without oedema or mass effect. Sometimes, microcysts (soap-bubble) may be seen within the mass ( Figure 2A ). About one-third of DNTs exhibit gadolinium enhancement. 15 Microscopically, the tumor is quite variable so that the differential diagnosis may be difficult in small specimens. Classically, there is a multinodular intracortical pattern of columns of oligodendroglial-like cells limiting a mucoid matrix with mature neurons (floating neurons) ( Figure 2B ). The surrounding cortex may harbor an alteration of the laminar structure probably due to a disorder of neuronal migration, configuring a true focal cortical dysplasia (FCD), considered in this case as FCD type IIIB by the International League Against Epilepsy (ILAE) scheme from 2011. 16 DNTs are grade1 tumors. Alterations in the *FGFR1* gene are found in the vast majority of DNTs. 17

### Pilocytic astrocytoma (PA)

**Figure 3:**
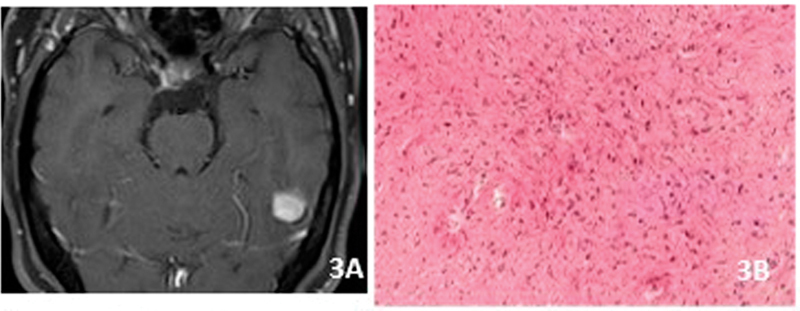
Pilocytic astrocytoma. A) Axial gadolinium Discrete enhancing lesion without oedema or mass effect. B) Piloid cells and Rosenthal fibres.

PA is the most frequent brain tumor in children. 18 Most are located in the cerebellum or diencephalic/optic pathways. As discussed above, from a strict point of view, they should not be considered a true LEAT for only occasionally a long-term history of epilepsy as the only clinical symptom is recorded in such tumors. Anyway, PAs are encountered in many series of surgeries for chronic epilepsy and are listed among other LEATs in more recent studies. 4 13 The typical MRI pattern is made of cyst with a gadolinium-enhancing mural nodule ( Figure 3A ). It is a benign astrocytic neoplasm (grade 1) with a classic biphasic histological appearance composed of more compact fibrillary tissues constituted by piloid bipolar cells, Rosenthal fibers and eosinophilic granular bodies, and a more loose, microcystic tissue with oligodendrocyte-like cells ( Figure 3B ). Most of PAs show alteration of MACK pathway with *KIAA1549:BRAF* fusion. However, this is more common in cerebellar specimens than in the lobar ones. 19

### Angiocentric glioma (AG)

**Figure 4:**
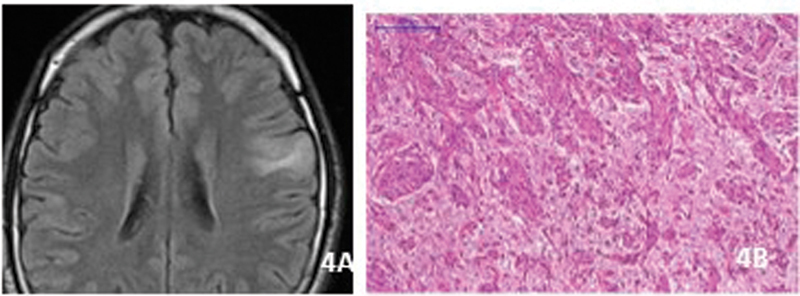
Angiocentric glioma. A) Axial FLAIR. Stalk-like extension of the tumor towards the ventricle. B) Rosette-like formation around vessels.

First recognized by Lelouch-Toubiana et al in 2005, 20 this neuroepithelial neoplasm is currently fully recognized as an anatomoclinical entity 21 being classified as a pediatric-type diffuse low-grade glioma by the last WHO Classification of brain tumors. 9 The vast majority of the cases occur in children and young adults. The tumors are located in the frontal, temporal, and parietal cortex. On MRI intratumoral T1 hyperintense areas and regional atrophy are commonly described. A stalk-like extension toward the adjacent ventricle is considered a distinctive sign 22 ( Figure 4A ), They are usually well-circumscribed, constituted by spindle cells radially oriented in a rosette-like pattern along vascular axes ( Figure 4B ). Almost all cases of AG harbor alterations of *MYB* gene. 23

### Diffuse astrocytoma, *MYB-* or *MYBL-1* altered

Many cases of this recently identified neoplasm of children or young adults, may have been in the past diagnosed as grade II diffuse astrocytomas. More recently, this tumor was referred to as “isomorphic diffuse glioma” 24 . However, contrary to low-grade astrocytomas in adults, they are IDH1 negative. Patients typically present with drug-resistant epileptic seizures. They occur in any cerebral lobe, more frequently in the temporal. On MRI, they are T1 hypointense, non-enhancing well-defined masses occupying cortical and sub-cortical areas with some degree of infiltration. 25 Histologically, there is diffuse infiltration of the neuropil by bland astrocytic cells without atypia or mitosis (grade 1). The diagnosis must be confirmed by molecular pathology, through the demonstration of alterations in *MYB* or *MYB1* genes. 26

### Polymorphous low-grade neuroepithelial tumor of the young (PLNTY)

**Figure 5:**
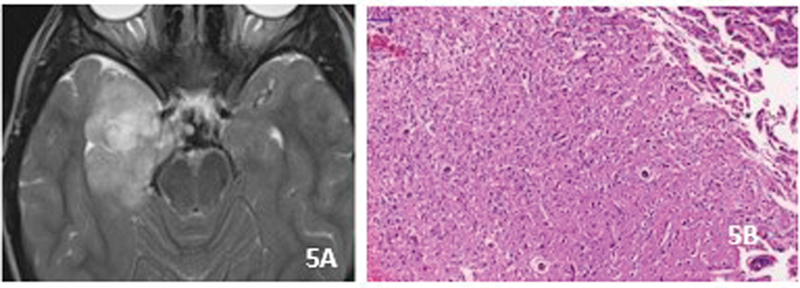
PLNTY. A) Axial FLAIR. Heterogeneity of hyperintense area. No mass effect or oedema. B) Diffuse proliferation of oligodendroglial-like cells.

Described by Huse et al. 27 in 2017 this LEAT is now recognized as an anatomoclinical entity. It occurs in children and young adults who present refractory epilepsy. Approximately, 80% occur in the temporal lobes. MRI shows cortical and subcortical components. Most lesions are solid-cystic hyperintense in T2 and FLAIR and hypointense in T1 ( Figure 5A ). Calcifications are very common. Histologically, oligodendroglial-like cells are the most frequent component. The association with calcification may make the distinction from oligodendrogliomas difficult ( Figure 5B ). However, the negativity for IDH1 and the frequent positivity for CD34 are helpful in defining the diagnosis. Atypia and mitosis are not seen. PLNTY is a grade 1 tumor. MAPK pathway abnormalities such as *BRAF ^V600E^* are frequently observed. 28

### Multinodular and vacuolating neuronal tumor (MVNT)

MVNT is considered a distinct tumor type in the 2021 WHO Classification. 9 Described by Huse et al in 2013 29 and identified in other series, this tumor occurs in adults whose main symptom is chronic epilepsy. Histologically, it is characterized by clusters of well-defined and coalescing nodules of neurons with vacuolar change of cytoplasm and matrix. Thus, it is considered a pure neuronal tumor (grade 1). MRI shows characteristic clustering of discrete or coalescent T2-FLAIR-hyperintense nodules in the deep cortex and superficial white matter. As with the other LEATs, there is a lack of mass effect or oedema. Similar to other low-grade glioneuronal tumors, alterations of the MAKP pathway have been demonstrated in this tumor. 30

## MANAGEMENT AND OUTCOMES

The long-term results of LEATs which underwent surgery are very satisfactory. As a whole, recurrence is a rare phenomenon as all anatomoclinical entities concerned are grade 1 tumors. As stated above, 13 most reports on recurrence or malignant transformation of such tumors lack genetic or molecular studies to exclude other diagnostic possibilities. Also, poor interobserver agreement on the diagnosis of these neoplasms, even among experimented neuropathologists is a well-known issue. 31 In a recent study, Delev et al. 32 claimed that LEATs bearing an activation of MACK-pathway and BRAF ^V600E^ mutation are associated with an increased risk for recurrence and malignant progression. From the clinical point of view, a favorable seizure outcome after surgery occurs in 75%-90% of the cases. 33 34 35 36 These studies show that factors linked to unfavorable seizure outcomes were older age at surgery, longer duration of epilepsy, acute post-operative seizures, partial extension of resection, and extra-temporal location of the neoplasm. So, the guidelines of an early intervention even in cases in which the seizures are well controlled with AED is the current rule in almost all neurosurgical centers. However, as LEATs are benign neoplasms and are expected to follow an indolent course, the need for adjuvant therapy (chemo or radiation therapy) for possible residual tumors is not recommended. A ‘wait and watch” approach must be considered, wherein additional intervention awaits the emergence of incontrovertible clinical or radiological progression. 37 This policy has been recommended by this author for pilocytic astrocytomas irrespective of their localization. 38

## RELATIONSHIP BETWEEN LEATs AND EPILEPSY

As stated in the initial paragraph of this manuscript, since the studies of Hughlings Jackson, the fact that any tumor located in the CNS can cause seizures is a well-known phenomenon. Indeed, the incidence of brain tumors in patients with epilepsy is about 4% and the frequency of epilepsy in patients with brain tumors is 30%. 39 However, patients with LEATs are more likely to develop chronic intractable epilepsy, which as stated above, is usually the only clinical manifestation. To explain the factor or factors implicated in the mechanisms of epileptogenesis, two main hypotheses have been proposed. 40 The tumor-centric hypothesis states that the epileptic activity derives from factors intrinsic to the tumor cells themselves. Aronica et al. 41 have demonstrated high expression of glutamate receptor (GluR) subtypes sustaining the hypothesis of a tumor component integrated into excitatory circuitries. More recently, Koh et al. 42 demonstrated the epileptogenicity of neurons transfected with BRAF ^V600E^ mutation in vivo. The epilepsy-centric hypothesis provides evidence that the infiltrated peritumoral cortex is key for tumor-related epileptic activity, due to metabolic imbalances of glioma-related glutamatergic and gamma-aminobutyric acid changes leading to epileptogenicity. Finally, the issue of the role of FCD associated wih some LEATs as GG and DNT (see above) is still matter of debate. As stated by Slegers and Blümcke 13 histopathology patterns of such FCD have never been scientifically defined, its prevalence varying by 25% to 75% of the cases. Also, as is the experience of the present author, there is an important intra and interobserver diagnostic non-concordance on these FCD. So, those authors expect that the ongoing molecular-genetic studies will help to clarify if these cases represent true FCD or pro-epileptogenic molecular interactions of the tumor with the surrounding peritumoral brain tissue.
